# Evaluation of internal margins for prostate for step and shoot intensity‐modulated radiation therapy and volumetric modulated arc therapy using different margin formulas

**DOI:** 10.1002/acm2.13707

**Published:** 2022-06-19

**Authors:** Daiki Higuchi, Tomohiro Ono, Ryo Kakino, Rihito Aizawa, Naoki Nakayasu, Hitoshi Ito, Takashi Sakamoto

**Affiliations:** ^1^ Department of Radiology Kyoto Katsura Hospital Kyoto Japan; ^2^ Department of Radiation Oncology and Image‐applied Therapy Kyoto University Graduate School of Medicine Kyoto Japan; ^3^ Kansai BNCT Medical Center Osaka Medical and Pharmaceutical University Osaka Japan; ^4^ Department of Radiation Oncology Kyoto Katsura Hospital Kyoto Japan

**Keywords:** internal margin for prostate, REML formula, step and shoot IMRT, van Herk formula, VMAT

## Abstract

**Purpose:**

This feasibility study evaluated the intra‐fractional prostate motion using an ultrasound image‐guided system during step and shoot intensity‐modulated radiation therapy (SS‐IMRT) and volumetric modulated arc therapy (VMAT). Moreover, the internal margins (IMs) using different margin formulas were calculated.

**Methods:**

Fourteen consecutive patients with prostate cancer who underwent SS‐IMRT (*n* = 5) or VMAT (*n* = 9) between March 2019 and April 2020 were considered. The intra‐fractional prostate motion was observed in the superior–inferior (SI), anterior–posterior (AP), and left–right (LR) directions. The displacement of the prostate was defined as the displacement from the initial position at the scanning start time, which was evaluated using the mean ± standard deviation (SD). IMs were calculated using the van Herk and restricted maximum likelihood (REML) formulas for SS‐IMRT and VMAT.

**Results:**

For SS‐IMRT, the maximum displacements of the prostate motion were 0.17 ± 0.18, 0.56 ± 0.86, and 0.18 ± 0.59 mm in the SI, AP, and LR directions, respectively. For VMAT, the maximum displacements of the prostate motion were 0.19 ± 0.64, 0.22 ± 0.35, and 0.14 ± 0.37 mm in the SI, AP, and LR directions, respectively. The IMs obtained for SS‐IMRT and VMAT were within 2.3 mm and 1.2 mm using the van Herk formula and within 1.2 mm and 0.8 mm using the REML formula.

**Conclusions:**

This feasibility study confirmed that intra‐fractional prostate motion was observed with SS‐IMRT and VMAT using different margin formulas. The IMs should be determined according to each irradiation technique using the REML margin.

## 1 INTRODUCTION

Prostate cancer is the leading cause of cancer‐related deaths worldwide.^1^, ^2^ Intensity‐modulated radiation therapy (IMRT) has become a standard treatment for localized prostate cancer.^3^, ^4^, ^5^ IMRT reduces normal‐tissue toxicity by sparing organs at risk without compromising the target dose. As a method of IMRT, step and shoot IMRT (SS‐IMRT) has found extensive use in current daily practices. Recently, volumetric modulated arc therapy (VMAT)—a more precise and advanced irradiation technique than IMRT, which uses the rotational therapy method—has been routinely used in many clinical facilities. VMAT has realized the delivery of highly conformal doses in shorter treatment times than conventional SS‐IMRT.

Although both SS‐IMRT and VMAT belong to the same category of IMRT, the large differences in durations of irradiation and subsequent effects on prostate movement, which are induced by rectal movement or bladder filling, during treatment sessions are worth noting.^6^ It is well known that prostate motion is highly dependent on treatment time.^7^, ^8^, ^9^, ^10^, ^11^ Ballhausen et al. evaluated the impact of intra‐fractional motion on treatment times of SS‐IMRT and VMAT. They also confirmed that shorter VMAT fractions reduced the displacement of prostate motion compared to longer SS‐IMRT fractions.^11^ According to the report by Ikeda et al., which evaluated intra‐fractional prostate motion using a detectable prostatic calcification with a kV X‐ray,^8^ the baseline drift was within 2.2 mm during a monitoring time of 15.9 min; furthermore, it was reported that the large baseline drift resulted in an inadequate dose coverage. In theory, the expected prostate motion is smaller in VMAT than in SS‐IMRT because the treatment delivery time of the former is shorter than that of the latter.^12^, ^13^ Pang et al. evaluated duration‐dependent margins for prostate motion for VMAT. They observed that the required margins were larger for a 15‐min treatment than an 8‐min treatment.^14^ However, to the best of our knowledge, no report has evaluated the REML margin for different irradiation techniques.

To manage the irradiation volume, inter‐fractional margins must be set to account for prostate motion during irradiation. Many studies have evaluated safety margins to account for set‐up errors and target motion.^8^, ^15^ The most commonly used margin formula was proposed by van Herk, and the calculation was performed using a systematic and random component of the uncertainty.^16^ However, this formula has a risk of overestimating systematic errors, as pointed out by Stroom et al. and Remeijer et al.^17^, ^18^ To resolve the overestimation of systematic errors by the van Herk formula, Matsuo et al. applied a variance component analysis to estimate the systematic and random errors.^19^ The analysis was proposed to estimate the population variance for systematic and random errors, as well as the population mean, based on an analysis of the variance. For the quantification of components, a variance component analysis using the restricted maximum likelihood (REML) formula was used. Ono et al. evaluated the intra‐fractional and inter‐fractional chest‐wall motions for breast radiotherapy during a deep inspiration breath‐hold, and the planning volume target (PTV) margin was estimated using the REML formula.^20^ They found that the PTV margin would be reduced using the REML formula excluding overestimation of systematic errors. Here, the PTV margin for prostate radiotherapy is highly dependent on imaging modality during treatment, the immobilization system, the irradiation technique (which have different treatment times), and the method of margin calculation; thus, the determination of an appropriate margin is required. If the margin can be reduced, the appropriate margins may contribute to the mitigation of adverse events without compromising the target dose.

The purpose of this study is to evaluate intra‐fractional prostate motion using an ultrasound image guidance system during SS‐IMRT and VMAT and to calculate the internal margin (IM) using different margin formulas under the same image‐guided radiotherapy (IGRT) condition, for identifying the REML margin for each irradiation technique.

## 2 MATERIALS AND METHODS

### 2.1 Patient population and data acquisition

**FIGURE 1:**
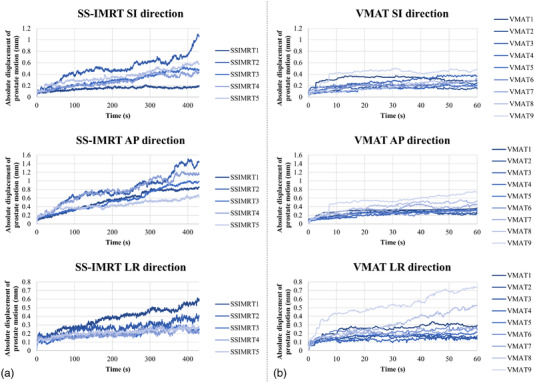
Schematic of the ultrasound image guidance device (TPUS: clarity autoscan transperineal ultrasound system) setup on a couch. The observation axes are shown as the superior–inferior (SI), anterior–posterior (AP), and left‐right (LR) directions

In total, 14 consecutive patients with prostate cancer, who underwent SS‐IMRT (*n* = 5) and VMAT (*n* = 9) between March 2019 and April 2020 at our institution, were included in this study. This study was approved by the Institutional Review Board of Kyoto Katsura Hospital. Table 1 shows details of patient characteristics. SS‐IMRT was performed on five patients (median age: 77 years, range: 69–84 years), and VMAT was performed on nine patients (median age: 70 years, range: 66–84 years). The prescribed dose was 74 Gy/37 fr, except for one case of VMAT with a dose of 60 Gy/20 fr. Computed tomography (CT) images were acquired by conventional scanning under free breathing using an OptimaCT 580 W system (GE Healthcare Technologies, Waukesha, WI). The CT slice thickness was 2.5 mm, and the CT images were captured in a field of view of 600 mm, on a 512 × 512 grid, at 120 kV and 100–335 mA. The BlueBag (Euromeditec, Tokyo, Japan) patient immobilization system was used to maintain reproducibility of the patient position. To observe prostate motion during MV beam delivering, the Clarity Autoscan transperineal ultrasound (TPUS) system (Elekta AB, Stockholm, Sweden) was used. Figure 1 shows a schematic of the initial setup of the Clarity Autoscan TPUS system. The Clarity Autoscan TPUS system is an image‐guided device that enables noninvasive, no‐radiation‐exposure, and real‐time observation of the prostate during irradiation.^21^ First, the reference prostate region of interest (ROI) was delineated by the radiation oncologist in our institution. After that, the reference prostate region of interest (ROI) data were input to the TPUS AutoScan probe, which optically tracked the center of the prostate ROI and allowed for 3‐dimensional image reconstruction and then set to the zero position. Prostate motion was observed in the superior–inferior (SI), anterior–posterior (AP), and left‐right (LR) directions. It should be noted that the TPUS system does not observe the prostate rotation.

**TABLE 1:** Information on patients to be studied

**Patient**	**Stage classification**	**Age**	**Gleason score**
SS‐IMRT1	cT2bN0M0	83	4 + 4
SS‐IMRT2	cT3aN0M0	77	4 + 4
SS‐IMRT3	cT1cN0M0	82	4 + 5
SS‐IMRT4	cT4N0M0	76	5 + 5
SS‐IMRT5	cT1cN0M0	72	3 + 3
VMAT1	cT2aN0M0	68	3 + 4
VMAT2	cT3aN0M0	73	4 + 5
VMAT3	cT1cN0M0	66	3 + 4
VAMT4	cT2aN0M0	70	3 + 4
VMAT5	cT2aN0M0	77	4 + 5
VMAT6	cT2aN0M0	71	4 + 4
VMAT7	cT2cN0M0	76	4 + 5
VMAT8	cT2aN0M0	84	3 + 4
VMAT9^a^	none	73	none

^a^ VMAT9 is palliative radiotherapy, so staging and Gleason score were not evaluated.

Abbreviations: SS‐IMRT, step and shoot intensity‐modulated radiation therapy; VMAT, volumetric modulated arc therapy.

### 2.2 Treatment planning system

Patients who underwent SS‐IMRT or VMAT were treated with the Vero4DRT system (Hitachi Ltd., Tokyo, Japan); SS‐IMRT plans were created using iPlan version 4.5 (BrainLab, Feldkirchen, Germany), and VMAT plans were created using RayStation version 6.2 (RaySearch Medical Laboratories AB, Stockholm, Sweden). As an intensity modulated technique, the treatment planning system of iPlan only supports the SS‐IMRT technique. The dose‐calculation algorithms were X‐ray voxel Monte Carlo (XVMC) version 4.1 for iPlan and Collapsed‐Cone Convolution (CCC) version 3.4 for RayStation, with a dose calculation grid size of 2.5 mm for both. SS‐IMRT was performed with seven fields of SS‐IMRT, and VMAT was performed with a 1–2 arc with a gantry angle sampling of 4° between the control points. Here, PTV was determined by adding a 7 mm margin for SI, LR, and the anterior directions and a 4 mm margin for the posterior direction to the clinical target volume.

### 2.3 Patient setup and prostate motion acquirement during beam delivering

For the treatment fractions of all patients, the intra‐fractional prostate motion was observed using the clarity TPUS system set on the treatment couch. Patients were aligned based on skin marks indicating the planning isocenter location with lasers mounted on the wall of the treatment room. For IGRT in SS‐IMRT and VMAT for prostate, a pair of orthogonal kV X‐ray images were obtained using the ExacTrac system (BrainLab, Feldkirchen, Germany) of Vero4DRT. The setup was aligned with the corresponding digitally reconstructed radiographs using the acquired images. Subsequently, cone beam CT (CBCT) images were scanned, and the final setup was aligned with the planning CT images based on the ROI of the prostate. If a significant amount of rectal gas was observed in the CBCT, gas venting was implemented and setup performed again. The initial position of the prostate was defined at the scanning start time and set as the zero position. Here, the observed time of prostate motion did not include setup and couch movement times. The treatment time was defined as the range from the beam irradiation start time to the beam irradiation end time. Thus, the prostate motion was observed during beam delivery.

### 2.4 Evaluation of prostate motion for IMRT and VMAT

The prostate motion was observed using the clarity TPUS system with a sampling rate of 4 Hz for the SI, AP, and LR directions. Prostate motions were observed in 37 fractions for 5 patients in SS‐IMRT, and in VMAT, prostate motions were observed in 37 fractions for 7 patients, 26 fractions for 1 patient, and 20 fractions for 1 patient. Subsequently, the prostate motions were compared between SS‐IMRT and VMAT using the Wilcoxon rank sum test. The statistical significance was set to *p* < 0.05. The maximum displacement of the prostate was defined as the displacement from the initial position of the prostate at the scanning start time. Here, no abnormal prostate motion was observed. In addition, the mean ± standard deviation (SD) was calculated from the maximum absolute displacement of all fractions for each patient.

### 2.5 Internal margin from van Herk formula

To calculate the IM of SS‐IMRT/VMAT for prostate cancer, two methods, namely the van Herk formula^22^ and the REML formula,^19^ were evaluated. Using the van Herk formula, the conventional definitions for systematic and random errors in the setup of radiotherapy are the SDs of the individual patient means and the root mean square of individual SDs, respectively.

Here, the IM was calculated using the van Herk formula^22^:

(1) $$2.5\sum_{\mathrm{eff}}{+0.7\sigma }_{\mathrm{eff}},$$

where ∑_eff_ and *σ*_eff_ are the effective values of the systematic and random errors, respectively. Instead of historical systematic and random errors by the van Herk formula, ∑_eff_ and *σ*_eff_ were used to consider the systematic effect of the random error under a small number of fractions or segments.^19^, ^23^, ^24^, ^25^ The effective values were used because this study included different fraction numbers and delivery techniques of SS‐IMRT and VMAT. For the van Herk formula, ∑*_eff_* is the quadratic sum of the standard deviation of all preparation (systematic) errors, and *σ_eff_* is the combined SD of treatment execution (random) variation. The coefficients of 2.5 and 0.7 were substituted to ensure that there was a 95% minimum dose to the clinical target volume for 90% of the patients covered. Using Equation (1), the IM of prostate motion was calculated for SS‐IMRT and VMAT.

### 2.6 Internal margin from the REML formula

Using the REML formula, the errors during beam delivery were evaluated by ∑_pt_, *σ*_fr_, and *σ*_intra_, which respectively represent the inter‐patient, inter‐fraction, and intra‐fraction SDs. Here, the errors were calculated from total prostate motion of all fractions in all patients. The overall mean was defined as the average value of the prostate motions of all fractions in all patients. A nested random‐effect model of the fraction of patients was adopted; that is, fraction levels were only meaningful within the levels of the patient. R version 4.0.0^26^ and lme4 package version 1.1‐23^27^ were used for the statistical analyses.

The ∑_eff_ and *σ*_eff_ values are defined as follows^19^, ^23^, ^24^, ^25^:

(2) $${\sum_{\mathrm{eff}}}^{2}={\sum_{\mathrm{pt}}}^{2}+\sigma_{\mathrm{fr}}^{2}/N,$$

(3) $$\sigma_{\mathrm{eff}}^{2}=\left(1-1/N\right)\;\sigma_{\mathrm{fr}}^{2}+\sigma_{\mathrm{intra}}^{2},$$

where ∑_pt_, *σ*_fr_, and *σ*_intra_ represent the combined values for the inter‐patient, inter‐fraction, and intra‐fraction SDs in the errors during beam delivery, respectively, and *N* represents the number of fractions. Using these equations, the systematic and random errors of the prostate motion were calculated, and the IM of prostate motion was derived from REML formulas using Equation (1) for SS‐IMRT and VMAT. In addition, 95% confidence intervals (CIs) were also evaluated.

## 3 RESULTS

### 3.1 Prostate motion and beam delivery time for SS‐IMRT/VMAT

**FIGURE 2:**
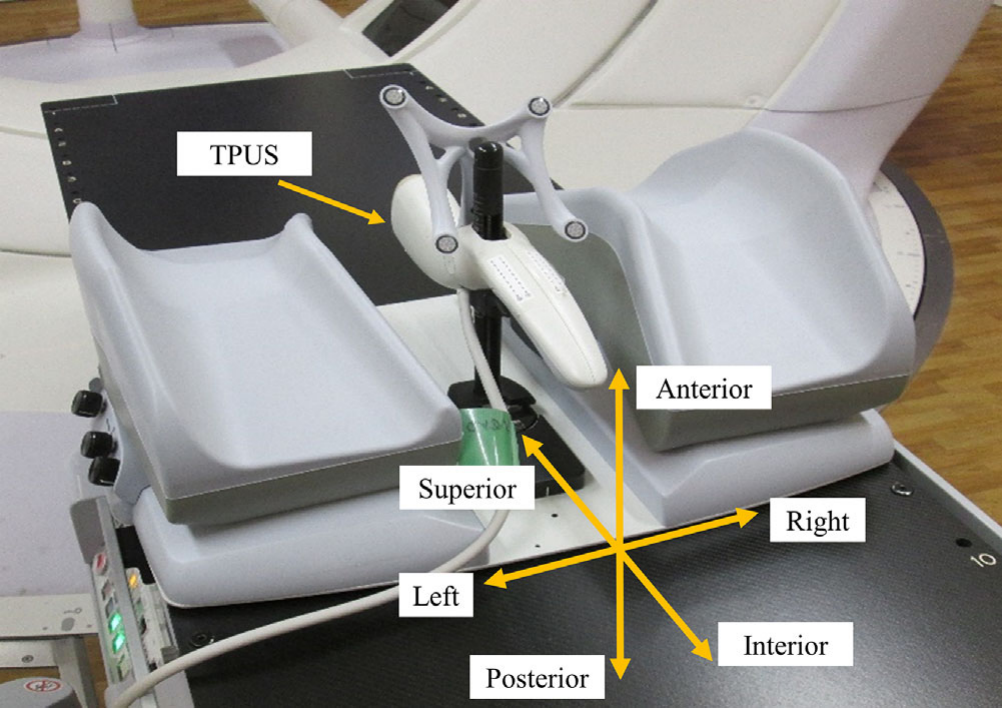
Absolute displacements of the prostate for (a) step and shoot intensity‐modulated radiation therapy (SS‐IMRT) and (b) volumetric modulated arc therapy (VMAT) in the superior–inferior (SI), anterior–posterior (AP), and left‐right (LR) directions. The initial position of the prostate was defined just before irradiation as zero displacement

**FIGURE 3:**
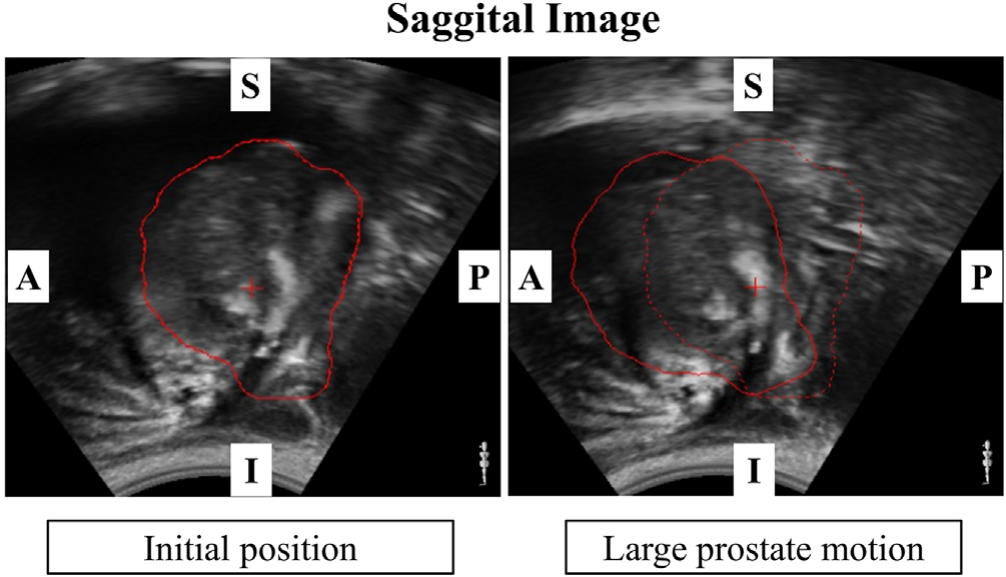
Example of an ultrasound image with one of the large prostate motions for volumetric modulated arc therapy (VMAT). Prostate suddenly moved in the AP direction owing to a rectal gas movement

**FIGURE 4:**
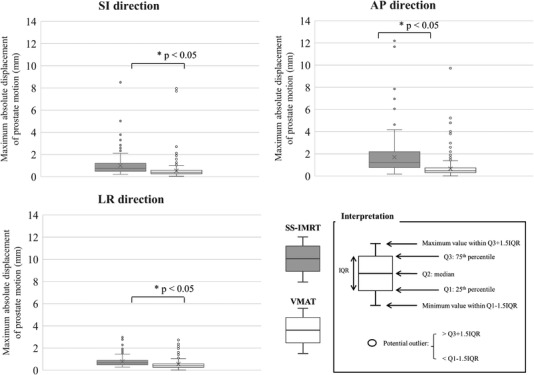
Variations in the maximum absolute displacement of the prostate between step and shoot intensity‐modulated radiation therapy (SS‐IMRT) and volumetric modulated arc therapy (VMAT)

The total number of radiotherapy sessions was 185 for the five SS‐IMRT patients and 305 for the nine VMAT patients. Average values of the planned monitor unit (MU) were 609.8 MU for SS‐IMRT and 483.3 MU for VMAT. Treatment times were 491.6 ± 54.2 s (minimum: 430.0 s, maximum: 567.0 s) for the SS‐IMRT and 78.7 ± 36.4 s (minimum: 60.0 s, maximum: 175.0 s) for VMAT. The treatment time for VMAT was shorter than that for SS‐IMRT. Figure 2a shows the average value of the absolute displacement of the prostate from the initial position for SS‐IMRT in the SI, AP, and LR directions as a function of time in seconds. In SS‐IMRT, the largest error was observed in the AP direction. Figure 2b shows the average value of the absolute displacement of the prostate from the initial position for VMAT in the SI, AP, and LR directions as a function of time in seconds. In VMAT, the largest displacement was observed in the AP direction. Figure 3 shows an example of an ultrasound image of one of the largest prostate motions. In this case, the prostate moved up to 9.7 mm in the AP direction owing to a sudden rectal gas movement. Figure 4 shows the variations in the maximum absolute displacement of the prostate in each patient between SS‐IMRT and VMAT. For SS‐IMRT, the maximum displacements of the prostate were 0.17 ± 0.18 mm (range: 0.22 to 8.50 mm), 0.56 ± 0.86 mm (range: 0.18 to 12.19 mm), and 0.18 ± 0.59 mm (range; 0.28 to 2.98 mm) for the SI, AP, and LR directions, respectively. For VMAT, the maximum displacements of the prostate were 0.19 ± 0.64 mm (range: 0.05 to 7.95 mm), 0.22 ± 0.35 mm (range: 0.02 to 9.72 mm), and 0.14 ± 0.37 mm (range: 0.03 to 2.73 mm) for the SI, AP, and LR directions, respectively. There were significant differences (*p* < 0.05) in prostate displacement between SS‐IMRT and VMAT in all directions, however, the differences were slight in the LR and the SI directions, and the difference was mainly only measurable in the AP direction.

### 3.2 Internal margin for SS‐IMRT/VMAT for prostate cancer

Table 2 lists the systematic and random errors of the prostate motion for SS‐IMRT and VMAT calculated using the van Herk formula. Here, the systematic and random errors used in the van Herk formula were comparable between SS‐IMRT and VMAT for the SI and LR directions. For the AP direction, the random error was larger in the cases treated with SS‐IMRT than in those treated with VMAT. The IMs from the van Herk formula were within 2.3 mm for SS‐IMRT and 1.2 mm for VMAT. Table 3 lists the systematic and random errors of the prostate motion for SS‐IMRT and VMAT calculated using the REML formula. Similar to the results of the van Herk formula, the systematic and random errors were comparable between SS‐IMRT and the VMAT for the SI and LR directions. The random error was larger for SS‐IMRT than for VMAT in the AP direction. The CIs of the overall mean did not significantly deviate from zero for both SS‐IMRT and VMAT. The IMs from the REML formula were within 1.2 mm for SS‐IMRT and 0.8 mm for VMAT. Comparing the two formulas, effective systematic errors from the van Herk formula were larger than that from the REML formula for all directions in each irradiation technique. Thus, the IM from the van Herk formula was larger than that from the REML formula for all directions. In addition, it was confirmed that the PTV margin of the current study fully encompassed the prostate motion.

**TABLE 2:** Deviation of prostate motion and internal margin for SS‐IMRT and VMAT calculated using the van Herk formula

	**SS‐IMRT**			**VMAT**		
	**SI**	**AP**	**LR**	**SI**	**AP**	**LR**
Σ_eff_ [mm]	0.11	0.30	0.12	0.16	0.20	0.12
σ_eff_ [mm]	1.34	2.26	0.89	0.82	0.98	0.99
IM [mm]	1.22	2.33	0.93	0.96	1.18	1.01

Abbreviations: AP, anterior‐posterior; IM, internal margin; LR, left–right; SI, superior–inferior; SS‐IMRT, step and shoot intensity‐modulated radiation therapy; VMAT, volumetric modulated arc therapy.

**TABLE 3:** Deviation of prostate motion and internal margins for SS‐IMRT and VMAT calculated using the REML formula

	**SS‐IMRT**			**VMAT**		
	**SI**	**AP**	**LR**	**SI**	**AP**	**LR**
Σ_pt_ [mm]	0.00	0.10	0.00	0.07	0.05	0.00
(95% CI [mm])	(0.00 to 0.09)	(0.00 to 0.30)	(0.00 to 0.08)	(0.00 to 0.17)	(0.00 to 0.16)	(0.00 to 0.13)
σ_fr_ [mm]	0.41	0.80	0.31	0.60	0.65	0.60
(95% CI [mm])	(0.37 to 0.46)	(0.73 to 0.89)	(0.28 to 0.35)	(0.56 to 0.65)	(0.60 to 0.71)	(0.56 to 0.65)
σ_intra_ [mm]	0.34	0.69	0.23	0.23	0.32	0.28
(95% CI [mm])	(0.34 to 0.34)	(0.69 to 0.69)	(0.23 to 0.23)	(0.23 to 0.23)	(0.32 to 0.33)	(0.28 to 0.28)
Overall mean [mm]	0.05	−0.10	0.03	0.07	−0.10	−0.07
(95% CI [mm])	(−0.01 to 0.11)	(−0.26 to 0.06)	(−0.01 to 0.08)	(−0.02 to 0.15)	(−0.18 to −0.01)	(−0.14 to −0.01)
Σ_eff_ [mm]	0.07	0.17	0.05	0.12	0.12	0.10
σ_eff_ [mm]	0.53	1.05	0.39	0.64	0.72	0.66
IM [mm]	0.54	1.15	0.40	0.75	0.80	0.71

Abbreviations: AP, anterior–posterior; CI, confidence interval; IM, internal margin; LR, left‐right; SI, superior–inferior; SS‐IMRT, step and shoot intensity‐modulated radiation therapy; VMAT, volumetric modulated arc therapy.

## 4 DISCUSSION

This study evaluated the intra‐fractional prostate motions for SS‐IMRT and VMAT using an ultrasound image guidance system.^21^ To the best of our knowledge, this is the first report to introduce a variance of the IM for prostate cancer that depends on the irradiation technique or margin calculation method as a feasibility study. Recently, hypo‐fractionated radiotherapy for prostate cancer has been widely applied.^28^, ^29^, ^30^ The IM setting is very important in such treatments because the number of fractions is reduced and the dose per fraction is larger. Furthermore, there has been growing interest in ultra‐hypo‐fractionated radiotherapy for prostate cancer.^31^ Our results contribute to clarifying appropriate IMs considering the systematic effect of the random error under a small number of fractions for prostate cancer radiotherapy, which allows us to calculate the REML margins for each treatment method and various number of fractions.

This study showed the variability in prostate motion for each irradiation technique and revealed that prostate motion varied with treatment time. Many studies have evaluated the motion of the prostate. Linda et al. observed prostate motion during treatment using pre‐treatment and post‐treatment CBCTs and reported that the average absolute value of prostate displacement was 1.5 mm in the AP direction, 1.0 mm in the SI, and 0.8 mm in the LR direction.^32^ ESTRO ACROP reported guidelines on the use of IGRT for localized prostate cancer.^15^ They reported that intrafraction prostate motion rigid shifts were to be 3–5 mm for a 5–10 min period. However, these studies used different irradiation and measurement devices. The current study evaluated prostate motion using the same measurement device for SS‐IMRT and VMAT and revealed that prostate motion was significantly larger in SS‐IMRT than in VMAT in all directions. Such time‐dependent variability in prostate position could be explained by physiological effects. Hamamoto et al. reported that large prostate motion was induced by abrupt rectal gas movement or bladder filling.^7^ Thus, considering that prostate motion depends on time, the IM should be considered in the treatment technique.

There are many reports on the required margins for prostate cancer. ESTRO ACROP recommended that PTV margins for the prostate may be in the range of 4–6 mm using on‐line correction.^15^ However, their report included various treatment techniques. Focusing on one irradiation technique, Sihono et al. evaluated intra‐fractional prostate motion during VMAT using transperineal ultrasound real‐time tracking in 38 prostate cancer patients.^33^ They calculated patient population‐based margins using the van Herk formula from the displacement of prostate motion and defined IMs of 1.25, 1.33, and 1.10 mm for the LR, AP, and SI directions, respectively. The current study observed an IM comparable to that reported by Shiono et al. for VMAT, obtained using the van Herk formula for prostate cancer. We confirmed that the use of the van Herk formula is correct in this study. As a new emphasized point in the current study, the IMs calculated using the van Herk formula were larger than those calculated by the REML formula for both the SS‐IMRT and VMAT irradiation techniques. This may be because the systematic errors were overestimated by the van Herk formula. Ono et al. reported internal and setup margins for breast radiotherapy during deep inspiration breath‐hold using the REML formula.^20^ They found that the systematic errors of the chest wall motion were smaller than the random errors, and appropriate systematic errors were indicated. In the current study, it was clarified that the appropriate IMs for the prostate were derived from the REML formula by excluding overestimation of systematic errors. Some studies have reported that a larger margin would increase the risk of late toxicity.^25^ In the current study, a small IM was obtained with the REML formula, which may contribute to mitigating late adverse events. However, our methods require further validation with a larger number of cases and institutions to confirm the results obtained in this study.

We acknowledge certain limitations of our study. First, owing to the small number of cases included in the current study, the range of CIs of the systemic and random errors for each method increased. A larger number of cases would lead to a narrower range of the CIs and a more appropriate margin. Second, few large prostate motions were observed in this study. In general, prostate motion is induced by rectal gas movement or bladder filling. Thus, it should be noted that the internal margin obtained from this study would be small because gas venting is implemented in our institution before beam delivery. To investigate the effect of rectal gas on the prostate, Shortall et al. evaluated the inter and intra‐fractional stability of rectal gas during magnetic resonance imaging guided radiotherapy and dose effects of rectal gas (MRIgRT).^34^ They estimated the volume of rectal gas in 174, 131, and 258 MRIs for six cervical, eleven bladder, and five prostate cancer cases. They observed that 60% of all patients would be delivered over an entire dose in the rectal wall owing to rectal gas, and the effect would be reduced by accounting for the daily adaptation. To implement a more appropriate treatment, intra‐fractional and daily adaptation or gas venting as per the current study are required.

## 5 CONCLUSIONS

This feasibility study confirmed that intra‐fractional prostate motion was evaluated using an ultrasound image guidance system for SS‐IMRT and VMAT. The IMs were larger for SS‐IMRT than for VMAT for each margin formula. An appropriate IM should be determined for each irradiation technique, and overestimation of systematic errors may be prevented using the REML formula. The appropriate margins may contribute to the mitigation of adverse events without compromising the target dose.

## CONFLICT OF INTEREST

The authors have no relevant conflicts of interest to disclose.

## AUTHOR CONTRIBUTIONS

Daiki Higuchi, Tomohiro Ono, Ryo Kakino, and Rihito Aizawa conceived the presented idea and verified the analytical methods. Naoki Nakayasu, Hitoshi Ito, and Takashi Sakamoto helped supervise the project. All authors discussed the results and contributed to the final manuscript.
